# The New York Institute of Stomatology

**Published:** 1903-04

**Authors:** 

**Affiliations:** The New York Institute of Stomatology


### Reports of Society Meetings.

### THE NEW YORK INSTITUTE OF STOMATOLOGY.

A meeting of the Institute was held at "The Chelsea," No. 222 West Twenty-third Street, New York, on Tuesday evening, March 3, 1903, the president, Dr. J. Morgan Howe, in the chair.

The minutes of the last meeting were read and approved.

Dr. Kimball, for the Executive Committee, stated that, owing to the illness of Dr. G. V. Black, the regular programme for the evening had been disarranged, but that the Institute expected to have Dr. Black's paper at a later meeting.

Dr. Bogue had been requested to present a paper which he had in the course of preparation.

Dr. Gillett presented to the meeting a brief report of his visit to Chicago to attend the meeting of the Odontographic Society.

*Dr. Gillett.*—Mr. President and Gentlemen,—It was a matter of very deep regret to me when I learned of Dr. Black's illness. I saw him in apparent good health only eight days before I learned that he could not be here.

While I hesitated at undertaking that very disagreeable duty of helping to fill a much larger man's place, I felt that possibly I might for a few minutes occupy your time interestingly with descriptions of a few of the good things I saw at Chicago last month.

The Odontographic Society's meeting clinic was by far the largest dental meeting the world has ever seen. When I arrived at the clinic room at about eleven o'clock of the first morning and asked for a programme, I was told that the entire supply of twelve hundred had already been given out. The next day one thousand more went the same way, and still many men were unable to get them. The attendance must have reached twenty-five hundred, and four hundred and fifty sat down to the banquet at the Auditorium Tuesday night. The banquet was worthy, the prearranged toasts were few, and good fellowship was everywhere in evidence.

The wonderful thing of this meeting was to see four hundred dentists all working tooth and nail to make their meeting the biggest ever known, every one of them putting aside his little jealousies, quarrels, and personal ambitions and working with a will for the common good. Patrick Henry said, "United we stand, divided we fall," and our forefathers fought it out on that basis. I say to you, "Divided we stand, united we progress." Later generations, nay, some of you, proved once more that "In Union there is Strength."

I want the privilege of quarrelling with my neighbor if I choose, but when it comes to furthering the interests of our profession, let's follow Chicago's lead, wipe the slate for the time at least, and close up shoulder to shoulder.

Dr. Gillett mentioned a number of appliances and methods that he had seen exhibited and demonstrated at Chicago.

He was especially pleased with the method for baking a porcelain crown for molar roots.

He was favorably impressed with a method for applying removable facings to bridges and crowns, called the Wedgelock tooth.

A new cataphoric appliance exhibited by Dr. Price was a marked advance over the earlier devices. By it the time required was reduced at least twenty-five per cent., and perhaps more. Dr. Price also exhibited an X-ray apparatus adapted for use in the dental office, that was a revelation to Dr. Gillett. It could be operated with much less noise than any apparatus he had previously seen.

The Coggswell crown and the Donaldson flask and press were among the appliances described and favorably commented on.

Dr. E. A. Bogue read a paper entitled "Some of the Causes of Irregular Teeth."

(For Dr. Bogue's paper, see page 241.)

DISCUSSION.

Dr. S. E. Davenport, in behalf of the Executive Committee, made his acknowledgments to Dr. Bogue for giving the Institute the paper to which all had just listened.

It seemed to Dr. Davenport that a knowledge of the principles that cause irregularity were at least being understood, even if orthodontists were not always agreed as to the proper way of correcting those irregularities. The Institute had had a rather remarkable series of presentations of the subject during the season, Drs. Angle and Baker, and now Dr. Bogue, having all given very scientific dissertations on the subject. Probably no busy practitioner in the world had given more attention to the subject than Dr. Bogue, or was so well prepared to prove his position by the presentation of casts extending over a long period of years. When Dr. Bogue presented a paper, all might depend upon it as being, not the result of impulse, but of years of practical study and observation.

Dr. Davenport thought all owed a great debt to Dr. Angle for calling attention in such emphatic language to the value of the sixth-year molar; and while this did not in any way detract from what Dr. Bogue had for years been teaching regarding the same matter, Dr. Angle's presentation of the scientific points of the subject was so complete that it had been a revelation to all. Dr. Davenport believed that Dr. Bogue himself had not thoroughly recognized the importance of all the points brought out in his own paper until recently, for his memory went back to a time a few years before, when he had heard Dr. Bogue state that upon the position of the lower cuspid teeth depended largely the ideal position of the teeth in the front of the arch.

Dr. Davenport called attention to that passage of Scripture, Matthew xxi., a part of the forty-second verse, which says, "The stone which the builders rejected, the same is become the head of the corner." At last dentists are beginning to realize that upon the retention of the sixth-year molar, over which there had been so many years of controversy, depended, more than upon any other tooth, the correct position of all the other teeth in the mouth.

Dr. Rolof B. Stanley agreed with Dr. Bogue that the first permanent molars are the keys to the situation. Malposition of the anterior teeth is a symptom, not a cause of the trouble. Dr. Stanley also asked whether the case where there was distal occlusion of the lower teeth on one side only was a mouth breather?

Dr. Bogue replied that he was.

Dr. L. C. Taylor, of Hartford, said that he had been very much pleased with the presentation of the illustrations. Dr. Taylor thought a great aid in cases of regulating was to give the mouth work to do. He had even advised his patients to chew gum; anything to give the teeth work and bring them into their proper articulation. A proper development of the masseter muscles was very beneficial. Work was what they needed in a great majority of cases.

The President pointed out that this was the first time that the necessity of bringing the upper and lower first molars into proper occlusion had been presented at any dental meeting, and that without waiting for the development of the other permanent teeth. He did not remember of ever hearing it before, and thought it a very important point.

Dr. C. O. Kimball had been struck very forcibly with the point made of regulating the teeth of young children, coming to us as they do, at the ages of five, six, seven, or eight. He had always been taught that we must wait until the teeth were all in before anything could be done in the way of correcting irregularities. Of course, there were certain things we could do while the teeth were developing, but in the main we must wait until such time as the teeth were fairly well erupted. We had all done this, and, with fairly good results and working along natural lines, had all made useful corrections. Dr. Brophy, of Chicago, working along a different line, had the credit of showing that during the developing period of the jaw was the time for making changes. Dr. Brophy had shown that in cases of separated palate this developmental period was the proper time for bringing the parts together, although it had been claimed by other surgeons that this could not be done until the process of development had been completed. It seemed to Dr. Kimball that Dr. Bogue was only carrying out this same idea in his paper to-night; that the key to the whole situation was the important point of taking these first molars, as soon as they are erupted, and placing them in their proper positions. Any little irregularities that came later, these two important teeth having been placed in proper position, could be comparatively easily corrected. He would like to ask Dr. Bogue the means he uses in placing these teeth in proper occlusion.

Dr. Bogue, in closing the discussion, stated that it was gratifying that so many men were sufficiently interested in the subject to stay and discuss it. He thanked Dr. Howe very cordially for seeing and acknowledging what he had hoped all would see,—that much of these detrimental irregularities can be prevented or corrected at a *very early age.* That was *the* essential feature and the one he intended to be brought out. Dr. Davenport was right,—we do grow as we work. These ideas had only gradually come to him, and the principles involved had crystalized into facts in the cases described by him in his paper read before the American Medical Association at Saratoga in May a year ago. He hoped, by and by, if he lived long enough, to be able to answer Dr. Kimball's questions better than he could do now. He did not know how to treat all children. He was very much indebted to Drs. Angle and Baker for their improvements in the original appliances. Some very simple apparatus, such as Dr. Angle uses, was as good as anything else, and far better than any complicated apparatus. Dr. Bogue presented, by illustration, a little apparatus, on similar lines, that was adapted for moving the first molars backward or forward, as well as for attaching all other necessary regulating devices, especially where Dr. Baker's reciprocally acting bands were applicable. This apparatus had no nuts or screws on the lingual sides of the rings, the attaching screws of the rings being hollow and serving all the purposes of the tubes, or so-called "pipes" used by Drs. Angle and Baker; and, having screw threads in their interior, they can also be utilized in other directions if needed.

Adjourned.

FRED. L. BOGUE, M.D., D.D.S.,
*Editor The New York Institute of Stomatology.*

